# Evaluating Changes in Cell-Wall Components Associated with Clubroot Resistance Using Fourier Transform Infrared Spectroscopy and RT-PCR

**DOI:** 10.3390/ijms18102058

**Published:** 2017-09-26

**Authors:** Rachid Lahlali, Tao Song, Mingguang Chu, Fengqun Yu, Saroj Kumar, Chithra Karunakaran, Gary Peng

**Affiliations:** 1 Canadian Light Source, 44 Innovation Blvd, Saskatoon, SK S7N 2V3, Canada; lahlali.r@gmail.com (R.L.); sarojgupta.k@gmail.com (S.K.); chithra.karunakaran@lightsource.ca (C.K.); 2 Currently Department of Crop Protection, Phytopathology Unit, Ecole Nationale d’Agriculture de Meknès, BP/S 40, Meknès 50001, Morocco; 3 Saskatoon Research and Development Centre, Agriculture and Agri-Food Canada 107 Science Place, Saskatoon, SK S7N 0X2, Canada; tao.song@agr.gc.ca (T.S.); chumg@163.com (M.C.); fengqun.yu@agr.gc.ca (F.Y.); 4 Currently Department of Biophysics, All India Institute of Medical Sciences, New Delhi 110029, India

**Keywords:** *Brassica napus*, callose deposition, infrared spectroscopy, phytoalexins, quantitative RT-PCR

## Abstract

Clubroot disease is a serious threat to canola production in western Canada and many parts of the world. *Rcr1* is a clubroot resistance (CR) gene identified recently and its molecular mechanisms in mediating CR have been studied using several omics approaches. The current study aimed to characterize the biochemical changes in the cell wall of canola roots connecting to key molecular mechanisms of this CR gene identified in prior studies using Fourier transform infrared (FTIR) spectroscopy. The expression of nine genes involved in phenylpropanoid metabolism was also studied using qPCR. Between susceptible (S) and resistance (R) samples, the most notable biochemical changes were related to an increased biosynthesis of lignin and phenolics. These results were supported by the transcription data on higher expression of *BrPAL1*. The up-regulation of PAL is indicative of an inducible defence response conferred by *Rcr1*; the activation of this basal defence gene via the phenylpropanoid pathway may contribute to clubroot resistance conferred by *Rcr1*. The data indicate that several cell-wall components, including lignin and pectin, may play a role in defence responses against clubroot. Principal components analysis of FTIR data separated non-inoculated samples from inoculated samples, but not so much between inoculated S and inoculated R samples. It is also shown that FTIR spectroscopy can be a useful tool in studying plant-pathogen interaction at cellular levels.

## 1. Introduction

Clubroot, caused by the obligate soil-borne pathogen *Plasmodiophora brassicae* Woronin, is a serious disease of Brassica crops worldwide [1] and a threat to canola (*Brassica napus* L.) production in western Canada [2]. An integrated approach [3] is recommended for managing the disease on canola, although host resistance is the cornerstone of the strategy. Clubroot resistance (CR) has been found from only limited sources, mainly *B. rapa* ssp. *rapifera* (turnips) and to a lesser extent *B. oleracea* L. [4,5,6]. So far, eight CR loci have been mapped to five *B. rapa* linkage groups [7,8,9,10], but the resistance mechanisms are generally unclear for most of these CR genes [11]. Some of the CR genes were incorporated into *B. napus* oilseed rape first in Europe; “Mendel” and “Tosca” were registered in 2000s [12], with each cultivar receiving one race-specific CR genes from a turnip cultivar. CR genes have also been incorporated into rutabaga (*B. napus*, ssp. *napobrassica*) [13], Chinese cabbage (*B. rapa* L.) [4,14], and canola [15]. Peng et al. [16] evaluated a wide range of Brassica germplasms and identified several highly resistant candidates against the predominant *P. brassicae* pathotype in western Canada. Further work characterized the CR gene *Rcr1* in *B. rapa* ssp. *Chinensis* [11].

Single-gene resistance to clubroot is often not durable; the breakdown of resistance has been widely reported on Chinese cabbage in Japan [17] and recently on canola in Canada [18]. Pyramiding or rotating multiple CR genes of different modes of action may improve the durability, but the effective use of such a strategy would require the knowledge of resistance mechanisms associated with specific CR genes. Such information is generally unavailable for most of the CR genes identified. A study based on the transcriptome analysis indicated that callose deposition is associated with the resistance conferred by *Rcr1* [11]. In separate studies on Arabidopsis, cell-wall constituents such as amino acids and phenylpropanoids were suggested to be related to clubroot resistance [19], while other host metabolic changes appeared to be associated with the pathogenicity of *P. brassicae* [20]. Many of these pathogen-induced metabolic changes, however, were suppressed in plants carrying *Rcr1* and such metabolic interference may include changes in the amount of fatty acids, phenolic or aromatic components, polysaccharides, and carbohydrates in root tissues. Some of these changes can be measured precisely with Fourier transform infrared (FTIR) spectroscopy, and when combining the data with molecular measurements during CR expression, it can be valuable information for understanding the cell-wall biochemical mechanisms associated with specific CR genes.

The FTIR spectroscopy is a powerful analytical technique capable of determining the biochemical composition of plant cells, especially for fatty acids, proteins, polysaccharides, and carbohydrates [21]. It is not known, however, whether it would identify the changes of these elements in canola roots during clubroot infection influenced by CR genes. The FTIR spectroscopy, with a spectral range from 4000 to 400 cm^−1^, is capable of analysing organic compounds containing −OH, −NH, and −CH functional groups [21,22] using a small sample size, therefore minimizing potential changes in the biochemical composition caused by sample processing during traditional cytological analyses. It is a procedure that provides a snapshot of biochemical composition in plant tissues or cells at specific points under diverse environments [21,22,23,24,25]. FTIR can generate a spectrum based on the vibrations of bonds within functional groups that can be considered as a biochemical or metabolic “fingerprint”. Based on the peak width, position, and the intensity of absorption, the configuration of molecular functional assemblies can be achieved [26,27,28]. FTIR may also be used for plant phenotyping based on signature biochemical elements [22], potentially complementing the selection based on genetic markers. The objective of this study was to assess the potential of using FTIR spectroscopy with quantitative PCR to identify biochemical and molecular traits associated with the clubroot resistance mediated by *Rcr1*, especially in relation to changes in cell wall components during the infection by *P. brassicae*, as suggested by the transcriptome study earlier [11].

## 2. Results and Discussion

### 2.1. Biochemical Changes in the Cell Wall

**Figure 1:**
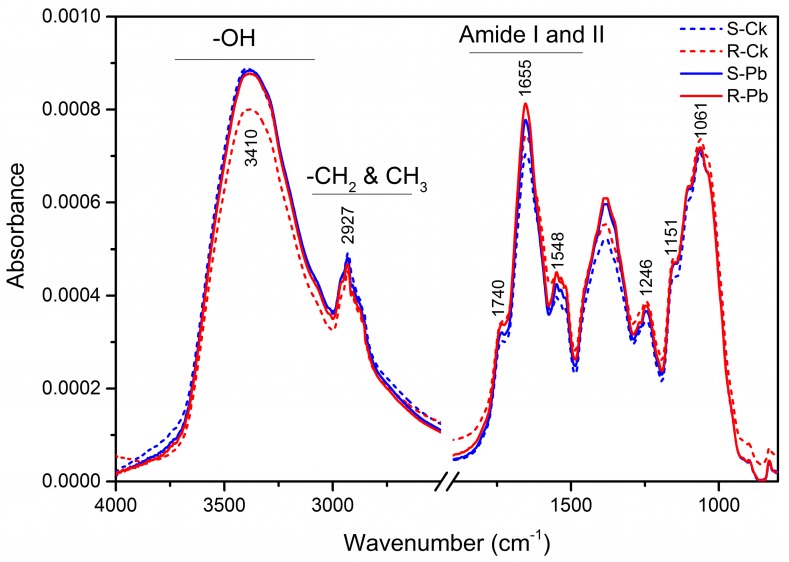
Mean infrared spectra of non-inoculated (Ck) and inoculated (Pb) samples of susceptible (S) and resistant (R) plants. Data were averaged over 20 pellets (10 per replicate) for each treatment.

The *P. brassicae* inoculation caused substantial changes in the spectra of resistant (R) and susceptible (S) roots when compared to respective controls; the characteristic spectral peaks that can be assigned to distinct functional groups are presented in Table 1 and Figure 1. Apart from the intensive but non-specific stretching bands for OH (3697–3098 cm^−1^) and alkyl C–H (~2924 cm^−1^) groups, the spectra showed two prominent peaks at 1655 and 1061 cm^−1^, which might be attributable to amide I (C=O stretch) and C–O–C vibrations, respectively, in cellulose (Table 1). The region from 3000 to 2700 cm^−1^ was dominated by the C–H stretching vibrations of –CH_3_, CH_2_, CH and CHO functional groups [29,30]. Some of the fingerprint regions showed intense peaks for carbonyl compound C=O groups (1740 cm^−1^), amide II (N–H stretch) and aromatic skeletal vibrations (1548 cm^−1^), methoxyphenolic substitutions in the aromatic unit of lignin (1518 cm^−1^), and –C–O/–C=O or P–O stretching (1151 cm^−1^), which is characteristic of carbohydrates, hemicelluloses, and phosphate [26,31,32,33].

**Figure 2:**
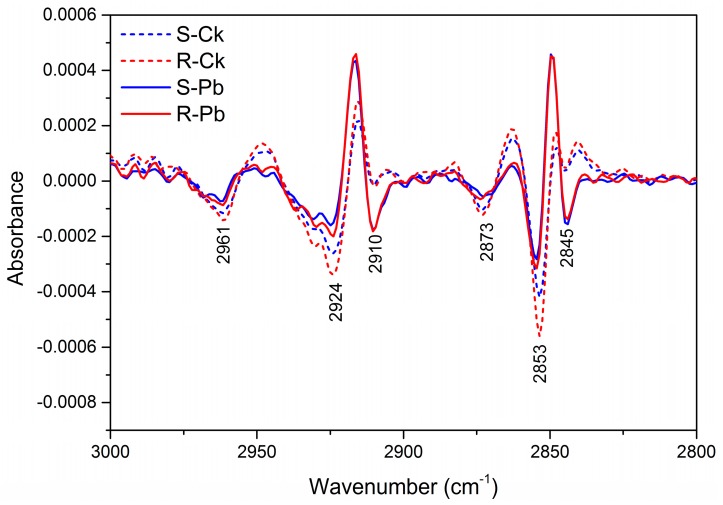
Absorbance of second derivatives in the lipid region (3000–2800 cm^−1^) for non-inoculated (Ck) and inoculated (Pb) samples of susceptible (S) and resistant (R) plants. Data were averaged over 20 pellets (10 per replicate) for each treatment. For secondary derivatives, dips represent the increase in chemistry; it goes in the opposite direction of spectral intensity.

**Figure 3:**
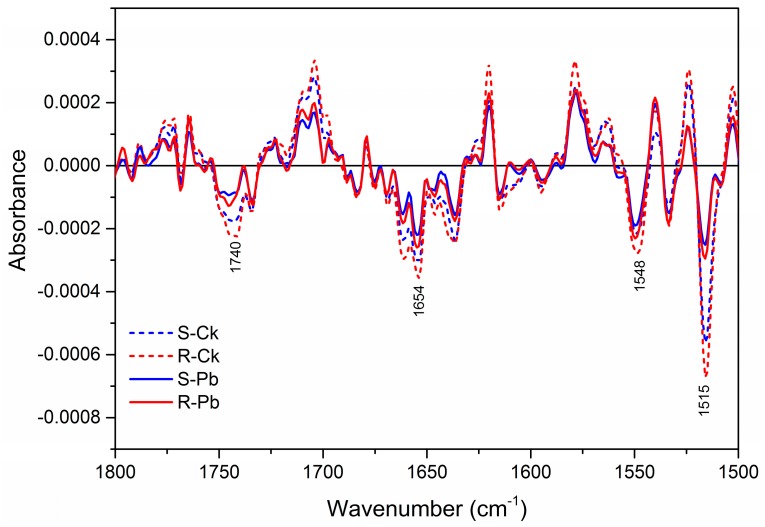
Absorbance of second derivatives in the fingerprint region (1800–1500 cm^−1^) between non-inoculated (Ck) and inoculated (Pb) root samples of susceptible (S) and resistant (R) canola plants. Data were averaged over 20 pellets (10 per replicate) for each treatment.

**Figure 4:**
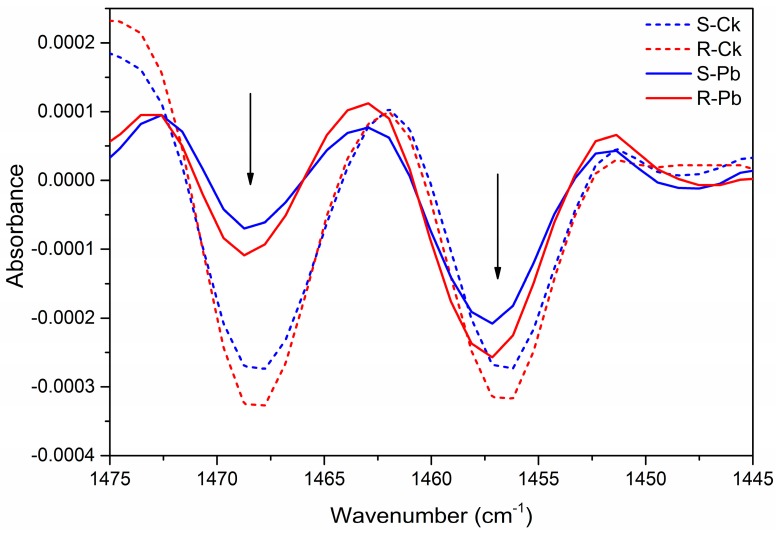
Absorbance of second derivatives in the fingerprint region (1500–1445 cm^−1^) between non-inoculated (Ck) and inoculated (Pb) root samples of susceptible (S) and resistant (R) canola lines. Data were averaged over 20 pellets (10 per replicate) for each treatment. Arrows indicate observable differences in chemistry between inoculated R and S samples.

The examination of secondary derivatives of respective absorbance also identified the differences among root samples (Figure 2, Figure 3 and Figure 4). There was a decrease in band intensity in inoculated R and S samples at 2961, 2924, 2873, and 2853 cm^−1^ relative to the respective non-inoculated controls, whereas the bands at 2910 and 2845 cm^−1^ increased (dips) in intensity in inoculated samples (Figure 2). This increase in two methylene bands may be used as markers for clubroot infection, and these changes seem to be correlated with a change in carbonyl esters at 1640 cm^−1^ (Figure 3) and a shift in the methylene bending vibration at 1475–1445 cm^−1^ (Figure 4), Based on the integrated absorption bands, phenolic groups (1580–1530 cm^−1^), lignin (1525–1505 cm^−1^), and pectin (840–815 cm^−1^) increased substantially in inoculated R samples relative to those in S samples (Table 2 and Table 3). Since the band in the amide I region can provide further insights into the protein secondary structure [34,35], we assessed the specific changes in secondary proteins and observed that the percentage of β-sheet was significantly higher in inoculated R than that in inoculated S samples (Table 4).This result may be of importance due to the many amino acids captured in this band (1628 cm^−1^), notably those implicated in resistance responses to plant diseases, especially l-phenylalanine; this amino acid is the key precursor for host defence metabolites produced via the phenylpropanoid and lignin pathways [36,37,38]. Lignin and phenolics (1620–1580 cm^−1^), as well as the carbonyl ester group (1740 cm^−1^) were substantially higher in inoculated R than inoculated S samples (Table 2 and Table 3) and this suggests that these compounds may contribute to clubroot resistance conferred by *Rcr1*.A small increase was observed in the symmetric and asymmetric stretching vibrations of PO_2_^−^ phosphodiestersand C–O stretching vibrations with inoculated S or R samples (Table 2). The 1200 to 800 cm^−1^ region is characteristic for the bands associated with C–C, C–O–C, and C–O–P stretching vibrations of polysaccharides [28,39,40].

PCA was performed on FTIR data from the lipid (3100–2800 cm^−1^) and fingerprint (1800–800 cm^−1^) regions to better understand the biochemical differences among samples. The results (PC-1 and PC-2) separated inoculated from non-inoculated root samples (59–97%), but did not differentiate between the inoculated R and S effectively.

This is the first report on using FTIR spectroscopy to understand plant-pathogen interaction based on changes in cell-wall components in conjunction with molecular traits linked to disease resistance. The study identified several characteristic bands associated with clubroot resistance that involve proteins, lignin, and phenolics (Table S1). These elements have been used in phenotyping plants [41,42], especially for the chemical profiling of the cell wall in response to biotic (fungal infection) and abiotic stresses [21,43,44]. Using the same approach, Lahlali et al. [45] identified chemical changes, particularly in lignin, cellulose, and hemicellulose, associated with wheat spikes infected by *Fusarium graminearum*. Martin et al. [46] used chemical fingerprints to discriminate disease resistant and susceptible clones of *Ulmus pumila*, and found spectral bands uniquely linked to two phenotypic groups [43,46]. Prior studies have used FTIR spectroscopy in combination with multivariate analysis to determine the changes in the metabolic patterns of tree roots before and after the infection by *Phytophthora cinnanoni* [47] or *P. Ramorum* [48]. This approach is considered simple, rapid, and cost-effective for high-throughput phenotyping as compared to other laboratory-extraction techniques [49,50] or molecular sequencing techniques [51].

### 2.2. Transcription of Cell-Wall and Lignin-Related Genes

Phenylpropanoids are involved commonly in plant defence responses [36], with lignin being induced as a physical barrier to infection [52]. l-Phenylalanine ammonia-lyase (PAL) is the entry-point enzyme in the phenylpropanoid pathway that can be induced by pathogens [41]; PAL is activated normally by an increased amount of reactive oxygen species (ROS), and is the key regulator in the phenylpropanoid pathway [53]. The FTIR results showed higher band absorption at 1720–1740 cm^−1^ for inoculated samples relative to that of non-inoculated controls (Figure 3), which is indicative of the oxidative stress associated with an increased ROS [34]. This shift in absorption intensity may be a by-product of lipid peroxidation caused also by oxidative stresses [34].

**Figure 5:**
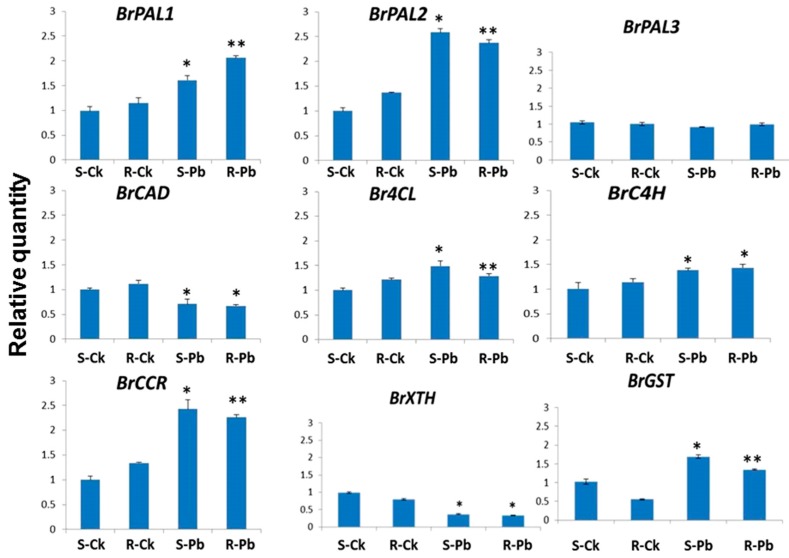
Expression of genes encoded for lignin biosynthesis [Phenylalanine ammonialyase (*BrPAL1*, *BrPAL2* and *BrPAL3*), 4-coumarate (CoA ligase-*Br4CL* and cinnamate-4-hydroxylase-*BrC4H*), hydroxycinnamoyl (cinnamyl alcohol dehydrogenase-*BrCAD* and cinnamoyl-CoA reductase-*BrCCR*) and cell-wall component (Glutathione-*S*-transferase-*BrGST* and Xyloglucanendo-transglycosylate-*BrXTH*)] in non-inoculated (S-Ck & R-Ck) and inoculated (S-Pb & R-Pb) susceptible (S) and resistant (R) root samples. Data were averaged over 6 replicates (3 technical × 2 biological replicates). *: Significantly higher or lower against the corresponding non-inoculated control (*p* < 0.05); **: Significantly higher or lower against the inoculated susceptible (S-Pb) samples (*p* < 0.05).

To better understand these biochemical changes, the expression of nine genes encoded for PAL biosynthesis and other cell-wall components was measured using qPCR. The gene *BrPAL1* showed substantially increased activity in root samples carrying *Rcr1* relative to that without the CR gene (Figure 5). The inoculation enhanced the expression of most genes involved in lignin biosynthesis, but the change was more pronounced with *BrPAL1* in roots carrying *Rcr1*, possibly increasing the lignification of the cell wall in these roots via the upregulation of lignin biosynthesis genes, especially *BrPAL1*. Zhao et al. [54] determined that lignin biosynthesis genes are upregulated with higher amounts of endogenous plant hormones, such as gibberellins. It is therefore possible that the regulation of lignin pathway by *Rcr1* can initially be through the activation of phenylpropanoid pathway, and this notion is supported by the result based on global transcriptome analysis that showed the involvement of cell-wall components in the clubroot resistance mediated by *Rcr1* [11]. The current transcript study was used to verify FTIR results by quantifying the expression of genes involved in lignin biosynthesis or cell wall synthesis/modification linked to resistance responses. The gene encoded for xyloglucanendo-transglycosylate (*BrXTH*) was down-regulated in inoculated samples (Figure 5). *BrXTH* can stimulate cell division, elongation, and plant growth [55,56], due partially to the accumulation of total Indole-3-acetic acid pool [55], but it does not appear that this event is strongly related to clubroot resistance/susceptibility. The key observation is likely the strong upregulation of *BrPAL1*, which encodes enzymes for the early biosynthesis of lignin. We have previously reported the importance of PAL in biocontrol of clubroot by several microbial agents, including *Bacillus subtilis*, *Heteroconium cheatospira* and *Colonosthys rosea* [57,58,59]. *BrPAL2*, *Br4CL*, *BrCCR* and *BrGST* showed slightly increased activities in inoculated S relatively to R samples (Figure 5). The *BrGST* is a family of multifunctional proteins encoding a family of genes ubiquitously found in bacteria, fungi, animals, and plants [60], and the expression of *BrGST* is commonly influenced by developmental processes, hormones, abiotic, and biotic stresses [61]. The slight up-regulation of these genes in inoculated S samples may suggest their involvement in infection and susceptibility.

The FTIR and qPCR analyses showed a connection between cell-wall strengthening and the up-regulation of *BrPAL1* mediated by *Rcr1*, and these mechanisms together contribute probably to clubroot resistance. Previous studies have attempted to define the major PAL peaks in aqueous solutions in the 1800–800 cm^−1^ region, with bands reported at 1628, 1583, 1528, 1448, and 1408 cm^−1^ as predominant peaks [62]. Coupled with a significant increase in the content of β-sheet (1628 cm^−1^) and bands encoding for phenolic and aromatic rings (1580–1215 cm^−1^), data from this study support the notion that PAL biosynthesis plays a role in plants carrying *Rcr1* in response to clubroot infection. Based on the biophysical and molecular analyses, it is further suggested that the activation of lignin and phenylpropanoid pathways is one of the mechanisms for clubroot resistance as conferred by *Rcr1*. Due to the complexity of the chemical composition in root tissues, FTIR data are usually considered qualitative rather than quantitative, which may allow to establish a pattern trend for compositional changes in plant cell wall under biotic and abiotic stresses [21]. Thus, caution is needed when interpreting FTIR data under different circumstances due to the sensitivity of technique; the results may be applicable only to specific conditions.

An integrated approach is required for clubroot management on canola [11,63,64], although host resistance is the cornerstone. For the optimal deployment of CR genes, it is useful to understand resistance mechanisms associated with specific CR genes. Recent attempts to characterize the CR gene *Rcr1* using transcriptome analysis [11] found the up-regulation of several genes involved in defence responses, including jasmonic acid/ethylene and callose deposition [11]. In this study, FTIR was used in combination with qPCR to analyse biochemical and molecular differences between S and R root samples, and identified that the PAL and lignin biosynthesis pathway are important to the clubroot resistance by this CR gene. This finding sheds further light on the resistance mechanisms of *Rcr1*; changes in cell-wall composition resulting from pathogen-induced lignification play a role.

Plant cell wall forms a dynamic physical barrier that often protects the cell from infection [65]. The chemical composition of cell wall, including cellulose, hemicellulose, and pectin vary among different tissues and can change during developmental, physiological, and pathological processes. The cell wall can also undergo dramatic structural and/or chemical changes under abiotic stresses [21,66], including lignification [67], callose deposition [68], protein cross-linking [69], accumulation of ROS, and phytoalexins [70]. FTIR can be a powerful tool to determine changes in the chemical composition of plant cell wall under biotic and abiotic stresses [21,34]. In the present study, infrared spectra were recorded in the mid-infrared range of 4000–600 cm^−1^ on bulk canola root samples, which may be used as a clubroot resistance finger-print region for *Rcr1*. It is hypothesized that additional finger-print regions may be identified with other CR genes if different resistance mechanisms are involved.

## 3. Materials and Methods

### 3.1. Plant Materials

An F_1_ population was developed by crossing the pakchoy (*B. rapa* ssp. *chinensis*) cv. Flower Nabana (Evergreen Y.H. Enterprises, Anahein, CA, USA), highly resistant to each of the five pathotypes of *P. brassicae* found in Canada [16], with a doubled haploid (DH) self-compatible susceptible canola (*B. rapa*) line developed at AAFC Saskatoon by using the protocol described previously [11,71]. Seeds were sown in the Sunshine #3 soil-less potting mix (SunGro Horticulture, Vancouver, BC, Canada) in tall plastic cones (5-cm diam., 20-cm tall) called “conetainers” (Steuwe& Sons, Corvalis, OR, USA), and plants were transplanted later into the same growth medium in 15-cm-diam. pots (1 plant/pot) at 5 about weeks after seeding. The planting mix was amended with 1% (*w*/*v*) 16-8-12 (N-P-K) control-released fertilizer, and all of the plants were kept in a growth room at 23/20°C (day/night) with a 14-h photoperiod (512 µmol/m^2^/s).

### 3.2. Pathogen Inoculum and Plant Inoculation

A field population of *P. brassicae*, consisting primarily of the predominant pathotype 3, was used for inoculation throughout the study. Mature clubroot galls filled with pathogen resting spores were dried at room temperature and stored at −20 °C until use. The inoculum was prepared as a resting-spore suspension using the method described previously [57], with the concentration adjusted to 1 × 10^7^ spores/mL. For inoculation, 5 mL of a resting-spore suspension were applied around the seed to result in about 1 × 10^6^ spores/g growth medium near the root zone. Inoculated plants were kept in the growth room and watered daily to maintain near-saturation soil moisture for two weeks to facilitate infection [72].

### 3.3. Root Sampling

Root samples were collected at 15 days post inoculation (dpi), when secondary infection is generally established in cortical tissue but clubbing symptoms are still not observed [73]. Samples were rinsed with tap water, and separated into inoculated susceptible (S-Pb) and resistant (R-Pb) groups based on the detection of *Rcr1* with the markers MS1-3 (5′-AAAACAAATATCCACCACG-3′ and 5′-CTCAATCCC-ACAAACCTG-3′) and A3-028 (5′-GAGGCCTCCTTTTCTGGTTT-3′ and 5′-CCGGAGAA-GTTT-GATTCGAG-3′). These markers are located at 24.02 and 25.36 Mb, respectively, on the chromosome A03 [11]. The effectiveness of these markers has been verified previously [11], and only the samples carrying *Rcr1* confirmed with both markers were kept for further testing. Non-inoculated susceptible (S-Ck) and resistant (R-Ck) roots were used as controls. The roots of nine random plants from each group were pooled as a biological replicate, with three replicates used for each group.

### 3.4. FTIR Spectroscopic Analysis

All of the infrared data were collected on a mid-infrared beamline (Canadian Light Source Inc., Saskatoon, SK, Canada) using the globar (silicon carbide) as the infrared source. The Bruker-IFS 66V/S spectrophotometer (Bruker Optics, Ettlingen, Germany) fitted with a deuteratedtriglycine sulphate (DTGS) detector was used for FTIR measurements. Root samples of S-Ck, R-Ck, S-Pb, and R-Pb were prepared using the protocol described earlier [45 with slight modifications. Root samples were dehydrated in a vacuum freeze drier (Labconco, Kansas City, MO, USA) and ground into fine powders with a mortar and pestle. Approximately 2 mg of freeze dried powder were homogenized with 0.93 g of dry potassium bromide (KBr) with a mortar and pestle, and made into a pellet under 8-ton pressure using a hydraulic press (Manual hydraulic press 15 Ton, Specac, Orpington, UK). Infrared spectrum was obtained in a transmission mode from each pellet, with KBr alone used as a background. Each IR spectrum was recorded in the mid infrared range of 4000–600 cm^−1^ wavenumbers at a spectral resolution of 2 cm^−1^. For each sample, the spectrum value was an average over 64 scans against the background spectrum value (KBr alone, 128 scans). Baseline correction was applied to each normalized spectrum using a rubber-band correction (64 points) and vectors normalized using the OPUS software (version 7.0, Bruker Optics Inc., Billerica, MA, USA). All FTIR spectra reported were averaged over two replicates (measurements of 10 pellets per replicate). The FTIR peaks reported in Table 1 were determined using the Quick Peaks routine in OriginPro with local max settings at 0% threshold height, no baseline, and the area at Y = 0 [34]. 

A second derivative was applied to the spectra to compare R and S samples and evaluate the effect of infection on the composition of the cell wall. The integrated absorption band area was determined using the OPUS integration method C [34]. The secondary protein structure of both R and S with/out inoculation was assessed following the protocol described by Lahlali et al. [34]. The Statistical Analysis System (SAS Institute, Cary, NC, USA) was used for data analysis. Analysis of Variance (ANOVA) and Least Significant Difference (LSD) were used to determine differences between inoculated and non-inoculated samples at *p* ≤ 0.05. Raw spectral data were imported into the Unscrambler software (Version 10.1; CAMO Software AS, Oslo, Norway) to extract the possible differences between R and S. Before the principal component analysis (PCA), spectral data were subjected to Multiplicative Scattering Correction (EMSC) and second derivatives to Goly-Savitzky algorithm using nine smoothing points [33]. PCA was performed on all datasets with each FTIR wavelength considered as an equally weighted variable [33].

### 3.5. Transcription of Cell-Wall and Lignin-Related Genes in Canola Root Samples

The objective of this experiment was to assess the transcriptional levels of selected genes involved in lignin biosynthetic pathways [Phenylalanine ammonialyase (*BrPAL1*, *BrPAL2* and *BrPAL3*), 4-coumarate (CoA ligase-*Br4CL* and cinnamate-4-hydroxylase-*BrC4H*), hydroxycinnamoyl (cinnamyl alcohol dehydrogenase-*BrCAD*, and cinnamoyl-CoA reductase-*BrCCR*), as well as other genes involved in cell wall metabolism (Glutathione-*S*-transferase-*BrGST* and Xyloglucanendo -transglycosylate-*BrXTH*) induced by the *P. brassicae* inoculation (Table S2) using a StepOne^®^ Plus quantitative RT-PCR (qPCR) system (Life Technologies, Mississauga, ON, Canada). RNA samples from both inoculated and non-inoculated roots were prepared, as described earlier [6]. Complementary DNA was synthesized using the Invitrogen Super Script III First-strand Synthesis system from 1 μg of total RNA, and PCR conducted using the Power SYBR green master mix (Life Technologies) following manufacturer’s instruction. Cycling conditions were 95 °C for the initial 10 min followed by 40 cycles of 15 s at 95 °C, 30 s at 50 °C, and finally 30 s at 60 °C.Melt-curve profiling and agarose gel electrophoresis were conducted to confirm the specificity of reaction and the absence of primer dimers. The actin gene Bra037560 was used as an endogenous control to normalize the expression level of target genes for its consistent level of expression among samples. The relative expression data were analysed using the StepOne^®^ software V2.2.2 (Life Technologies).The whole transcription experiment was run twice (two biological replicates), with three technical replicates for each of the sample groups in a repetition. ANOVA and LSD (*p* < 0.01) were performed using the software Statistical Product and Service Solutions (V20.0; IBM, Markham, ON, Canada) for transcription quantity with each of the genes examined.

## 4. Conclusions

The current study characterized several biochemical and molecular changes in canola roots associated with the CR gene *Rcr1* in response to clubroot infection using FTIR and qPCR analyses. The most relevant changes appear to be related to the increased biosynthesis of lignin and phenolic rings. These results were generally in agreement with the transcriptome data published previously on the higher expression of genes encoded for callose deposition. The up-regulation of PAL is indicative of an inducible defence response by *Rcr1*; the activation of a basal defence gene like *BrPAL1* through the phenylpropanoid pathway, and lignin accumulation may contribute to the clubroot resistance. The FTIR data indicate that cell-wall components may play a role in defence responses against clubroot mediated by *Rcr1*. This work also shows the value of using FTIR spectroscopy in conjunction with molecular tools in studying plant-pathogen interactions.

## Figures and Tables

**Table 1:** The assignment of main functional groups based on Fourier transform infrared (FTIR) spectra [21,26,32,33] of susceptible (S) and resistant (R) root samples. Wave numbers presented in the table are the mean of vibrational range.

Wavenumber (cm^−1^)	Absorption Peak Location and Assignment	Components
3410	–OH groups, NH stretching	Proteins (Amide A)
2927	C–H stretch (asym.) of CH_2_	Lipid acyl chains
1740	C=O stretching: carbonyl ester compounds	Pectin
1650	–C=O– and –C=N– stretching	Proteins (Amide I)
1548	N–H bending and C–N stretching	Proteins (Amide II)
1518	CH_2_ and CH_3_ methylene chain stretching	Lignin
1246	C–O, –CH_2_- stretching and bending, P–O stretching	Hemicellulose
1151	C–O–C asymmetric stretching, PO_2_ stretching	Cellulose
1061	C–O–C symmetric stretching	Cellulose

**Table 2:** Integrated absorption band area of –OH stretching, amide A (3680–3000), carbonyl ester (1760–1720 cm^−1^), amide I (1700–1620 cm^−1^), amide II (1620–1580 cm^−1^) and phenolic groups (1580–1530 cm^−1^).

	Amide A	Acyl Lipids	Carbonyl Ester	Amide I	Amide II	Phenolic Ring
S-CK	564.0 ± 7.4a	34.7± 1.5ab	10.8 ± 0.5ab	61.8 ± 1.9a	14.7 ± 0.7b	4.1 ± 0.2a
S-Pb	620.3 ± 8.7c	36.1 ± 1.1b	10.7 ± 0.5ab	68.6 ± 1.7c	15.2 ± 0.7b	5.3 ± 0.3b
R-CK	597.0 ± 8.8b	35.6 ± 1.1ab	9.9 ± 0.5a	63.8 ± 1.6ab	15.0 ± 0.6b	4.1 ± 0.2a
R-Pb	550.9 ± 9.5a	33.7 ± 1.1a	11.0 ± 0.4b	65.7 ± 1.9bc	13.6 ± 0.5a	6.1 ± 0.2c

R: Resistant canola line carrying *Rcr1*; S: Susceptible line without *Rcr1*; Ck: non-inoculated; Pb: Inoculated with *Plasmodiophora brassicae*. Means in the same column followed by the same letter do not differ (Least significant difference (LSD), *p* = 0.05).

**Table 3:** Integrated absorption band area for lignin (1525–1505 cm^−1^), hemicellulose (1275–1215 cm^−1^), cellulose (1090–1043 cm^−1^), and ring vibration of pectin (840–815 cm^−1^) insusceptible (S) and resistant (R) samples under non-inoculated (Ck) and inoculated (Pb) conditions.

	Lignin	Hemicellulose	Cellulose	Pectin
S-CK	3.3 ± 0.2a	8.3 ± 0.2a	62.8 ± 2.8b	0.8 ± 0.0a
S-PK	3.7 ± 0.1b	8.7 ± 0.2b	60.7 ± 1.5b	0.8 ± 0.1a
R-CK	3.2 ± 0.1a	8.2 ± 0.2a	64.0 ± 2.4b	0.8 ± 0.1a
R-Pb	4.0 ± 0.1c	8.7 ± 0.2b	55.3 ± 2.5a	0.9 ± 0.1b

R: Resistant canola line carrying *Rcr1*; S: Susceptible line without *Rcr1*; Ck: non-inoculated; Pb: Inoculated with *P. brassicae*. Means in the same column followed by the same letter do not differ (LSD, *p* = 0.05).

**Table 4:** The percentage of secondary protein structures found in resistant (R) and susceptible (S) samples (*n* = 20).

	Non-Inoculated (Ck)		Inoculated (Pb)	
	S	R	S	R
β-sheet (PAL) ^1^	46.4c ^2^	44.2b	43.5a	48.3d
α-helices	24.2c	22.0b	26.0d	21.5a
Others	29.5a	33.8d	30.4c	30.2b

^1^ PAL: phenylalanine ammonia lyase; ^2^ Means in the same row followed by the same letter do not differ (LSD, *p* = 0.05).

## Supplementary Materials

Supplementary materials can be found at www.mdpi.com/1422-0067/18/10/2058/s1.

## Abbreviations

FTIR	Fourier transform infrared spectroscopy
qRT-PC	Quantitative PCR
CR	Clubroot resistance
PCA	Principal component analysis
R	Resistant
S	Susceptible
Pb	*Plasmodiophorabrassicae*
Dpi	Days post-inoculation
