# Supplementary material for: Evaluating Changes in Cell-Wall Components Associated with Clubroot Resistance Using Fourier Transform Infrared Spectroscopy and RT-PCR
Source: Int J Mol Sci. 2017 Sep 26;18(10):2058. doi: 10.3390/ijms18102058 (PMC5666740; doi:10.3390/ijms18102058)
Supplement: Supplementary file 1 [file ijms-18-02058-s001.pdf]

Table S1. Spectral interpretation and assignment of canola root samples after multivariate analysis.

Wavenumber (cm^{-1})	Assignment	Group
1655	C-N stretching amide I	Proteins
1578	N-H deformation amide II	Proteins
1553	N-H deformation amide II	Proteins
1542	N-H deformation amide II	Proteins
1520	Skeletal bands of aromatic rings	Lignin
1483	C-H bending in alkyl group	Proteins
1467	CH2 bending of methylene chains in lignin	Lignin
1351	CH2 bending	Xyloglucan, cellulose
1275	C-O stretching	Lignin, pectin
1261	C-O stretching	Pectin
1250	C-O stretching	Pectin/lignin

Table S2. Primer sequences used for quantitative real-time PCR analysis.

Gene Name	Accession No.	Forward	Reverse	Reference
BrPAL1	DQ341309	CAAAGCGATTCACGGAGGTAA	CGCTCCTTTGAAACCGTAGTC	[58]
BrPAL2	AY795080	TTGGATTACGGATTCAAAGGA	CGAGATGAGTCCCAAAGAGTT	[58]
BrPAL3	AY055752	AGAACGGTGTCGCTCTTCAG	TGTGGCGGAGTGTGGTAATG	[58]
BrC4H	DQ485129	TGACTTTAAGTATGTGCCGTTTG	GGACCTTGGCTTCATTACGAT	[58]
Br4CL	CX190902	TAATCCGAATCTTTACTTCCACAG	GCAACCGTCACTTTACACCTCT	[58]
BrCCR	CD844319	GGTGGAAGTTTAGGTCATTAGAAGA	CCAATAGTAGACTTGAGGAGGTGAA	[58]
BrCAD	CD814354	TTATGTCCTGGTTGGTTTCCC	CACCCTTTCAATAGCTTCGTTT	[58]
BrGST	AI352707	GTGGCTGAGATCACCAAGAG	TACTGCGACTGAAGCAGAAG	This work
BrXTH	AY156708	GTCCGCATGAAATGGACCATCTAC	CCTTCTACATTCGGCTGGCATAAC	This work
